# Design and Implementation of a Novel Polarimetric Active Radar Calibrator for Gaofen-3 SAR

**DOI:** 10.3390/s18082620

**Published:** 2018-08-10

**Authors:** Liang Li, Yongtao Zhu, Jun Hong, Feng Ming, Yu Wang

**Affiliations:** 1 Institute of Electronics, Chinese Academy of Sciences, Beijing 100190, China; zyt@mail.ie.ac.cn (Y.Z.); junhong@mail.ie.ac.cn (J.H.); mingfengmail@sina.com.cn (F.M.); wangyu@mail.ie.ac.cn (Y.W.); 2 National Key Laboratory of Sciences and Technology on Microwave Imaging, Beijing 100190, China; 3 Graduate University of Chinese Academy of Sciences, Beijing 100049, China

**Keywords:** GF-3, transponder, polarimetric active radar calibrator, SAR calibration

## Abstract

The Chinese first fully polarimetric space-borne synthetic aperture radar (SAR)-Gaofen-3 (GF-3) was launched in August 2016, which operates at the C-band and the resolution can reach 1 m. Polarimetric SAR calibration is a procedure that corrects the polarization distortion of a measured scattering matrix by referring to the scattering matrix of a known target. The present paper describes the principle, design, manufacture, and measurement results of a novel polarimetric active radar calibrator (PARC) designed for GF-3. A new design method for PARC was presented and two dual-polarized antennas with very high polarization purity were used. The internal calibration technique was introduced to ensure balance in the amplitude and phase, which ensures the precision of the PARC’s scattering matrices. The results we obtained through measurement in the microwave anechoic chamber and experiments in in-orbit calibration agree well with the theoretical predictions, and the novel PARC presented is proved to be well suited for polarization and radiometric calibration of GF-3.

## 1. Introduction

The Chinese satellite, Gaofen-3 (GF-3), was launched in August 2016. It is the first satellite of China equipped with a fully polarimetric synthetic aperture radar (SAR), which operates at the C-band and its highest resolution can reach 1 m [1]. Tens of nominal operation modes, such as StripMap mode, Spotlight mode, ScanSAR mode, globe observation mode, ultra-fine mode, wave imaging mode, and so on, are available [2,3,4]. Therefore, GF-3 can be used in many fields, such as disaster monitoring, meteorological observation, marine monitoring, global environmental studies, and so on. As China’s first fully polarimetric spaceborne SAR, the GF-3 has three fully polarimetric SAR imaging modes, therefore, polarimetric calibration is very important and necessary for GF-3.

To achieve a higher precision of the GF-3 SAR, a detailed in-orbit calibration plan was established and a calibration campaign has been carried out. Compared to conventional single polarimetric SAR, the fully polarimetric SAR can obtain a variety of polarimetric information of targets. Additionally, it provides a wide aspect of targets with a scattering matrix. When the scattering matrix of a target is measured with the fully polarimetric SAR, it usually gets distorted due to the imperfect polarization transfer characteristics of the SAR’s transmitting and receiving antennas. This polarization distortion in the measured scattering matrix must be removed before the data can be used in several polarimetric applications. This procedure is called polarimetric calibration [5,6].

In polarimetric calibration, the reference target is measured with the polarimetric SAR being tested, and the measured scattering matrix is analyzed to estimate the polarization transfer characteristics of the SAR by referring to the known scattering matrix of the reference target. Polarimetric active radar calibrators (PARC) are usually needed in polarimetric calibration because it can provide different scattering matrices. There are a variety of methods for implementing the PARC at present. The PARC used by Freeman et al. [7] was a typical depolarizing target in which the polarization directions of the receiving and transmitting antennas were orthogonal to one another, but it can only provide the scattering matrix of [0 0; 1 0] or [0 1; 0 0]. Sarabandi et al. [8] developed a single antenna PARC (SAPARC) that used a dual-polarized horn antenna for both transmission and reception with an ortho-mode transducer (OMT) to separate the transmitting and receiving signals. The SAPARC was used for SIR-C calibration [9]. However, to reach a better polarimetric isolation, the antenna length including the OMT appears to be about 2–3 m and it is difficult to handle and point at an SAR satellite in the field. Again, the SAPARC can only provide the scattering matrix of [0 0; 1 0] or [0 1; 0 0]. The PARCs used for Radarsat-2 and Sentinel-1 are realized through the rotation of the receiving antenna and the transmitting antenna. However, the scattering matrices cannot be set flexibly for one PARC. The PARC of the Kompsat-5 mission, realized using four antennas, including a H-polarized receiving antenna, V-polarized receiving antenna [10,11], H-polarized transmitting antenna, and V-polarized transmitting antenna, had scattering matrices that could be set flexibly for each PARC [12]. However, this structure is large and probably heavy due to the four antennas, and it is, therefore, difficult to handle and point this large structure towards an SAR satellite in the field.

To overcome these problems, the author proposed a novel PARC that used a dual-polarization receiving antenna and a dual-polarization transmitting antenna, which can provide the scattering matrices, such as [0 0; 1 0], [0 1; 0 0], [−1 −1; 1 1], and [1 0; 0 1]. Five novel PARCs were developed for the GF-3 SAR calibration and they have been used to implement the calibration campaign during the first three months since launch successfully.

## 2. Principle of Polarimetric Calibration

Neglecting the noise, the polarimetric measurement mathematical model in a practical SAR system is simplified by [13,14]:

(1) $$M=Ae^{i\phi }\left(\begin{array}{cc} 1 & \delta_{1}\\ \delta_{2} & f_{1} \end{array}\right)\left(\begin{array}{cc} S_{hh} & S_{hv}\\ S_{vh} & S_{vv} \end{array}\right)\left(\begin{array}{cc} 1 & \delta_{4}\\ \delta_{3} & f_{2} \end{array}\right)=Ae^{i\phi }RST$$

where $M=[M_{hh}\;M_{hv};\;M_{vh}\;M_{vv}]$ and $S=[S_{hh}\;S_{hv};\;S_{vh}\;S_{vv}]$ are the measured and theoretical scattering matrix of the target, respectively. *R* and *T* are the SAR receiving and transmitting distortion matrices, respectively. *A* represents the SAR system gains and losses, and *φ* is any phase shift due to the round-trip delay between the target and SAR. *f*_1_ and *f*_2_ are the channel imbalances between the H and V channels when receiving and transmitting, respectively. *δ*_1_ and *δ*_2_ are the cross talks when a wave is received. *δ*_3_ and *δ*_4_ are the cross talks when a wave is transmitted.

**Figure 1:**
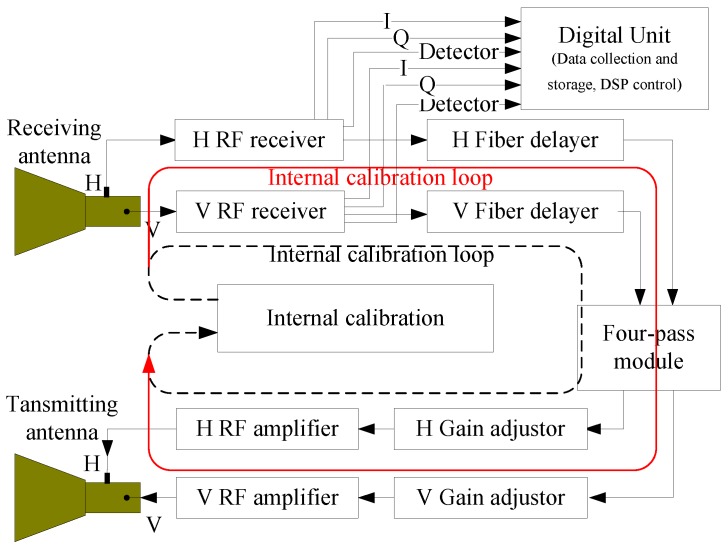
System block diagram of the GF-3 transponder.

It can be seen from Equation (1) that the theoretical scattering matrix of the target can be derived from the measured scattering matrix if *Ae^jφ^*, *R*, and *T* can be estimated for a given SAR image. An approach that has been developed for estimating *R* and *T* is to use a combination of three PARCs with the scattering matrices, [0 0; 1 0], [0 1; 0 0], and [−1 1; 1 1] [5,6]. The PARC developed for the GF-3 consists mainly of two dual-polarization antennas, an radio frequency (RF)receiver, fiber delayer, four-pass module, gain adjustor, RF amplifier, internal calibration module, and so on. The system block diagram is shown in Figure 1.

From Figure 1, we can see that the PARC can provide the scattering matrix, [0 0; 1 0], when only the V RF receiver, V fiber delayer, H gain adjustor, and H RF amplifier are open and the switch of V-H is open in the four-pass module; [0 1; 0 0] when only the H RF receiver, H fiber delayer, V gain adjustor, and V RF amplifier are open and the switch of H-V is open in the four-pass module, [−1 −1; and 1 1] when the H RF receiver, V RF receiver, H fiber delayer, V fiber delayer, V gain adjustor, H gain adjustor, V RF amplifier, and H RF amplifier are all open and the switches of H-H/V and V-H/V are open in the four-pass module. Now, the three scattering matrices used for polarimetric calibration can all be provided by the PARC we developed. Furthermore, the scattering matrix, [1 0; 0 1], can also be obtained from the PARC when the H RF receiver, V RF receiver, H fiber delayer, V fiber delayer, V gain adjustor, H gain adjustor, V RF amplifier, and H RF amplifier are all open and the switches of H-H and V-V are open in the four-pass module. This matrix can be used to verify the result of the polarimetric calibration.

Until now, we can see the PARC developed for the GF-3 not only can provide the scattering matrices, [0 0; 1 0], [0 1; 0 0], and [−1 −1; 1 1], used for polarimetric calibration, but also the scattering matrix, [1 0; 0 1], used to verify the calibration result, which never happened in previous PARC, such as Radarsat-2, Sentinel-1, TerraSAR, and so on.

## 3. Design and Implementation of the PARC

**Figure 2:**
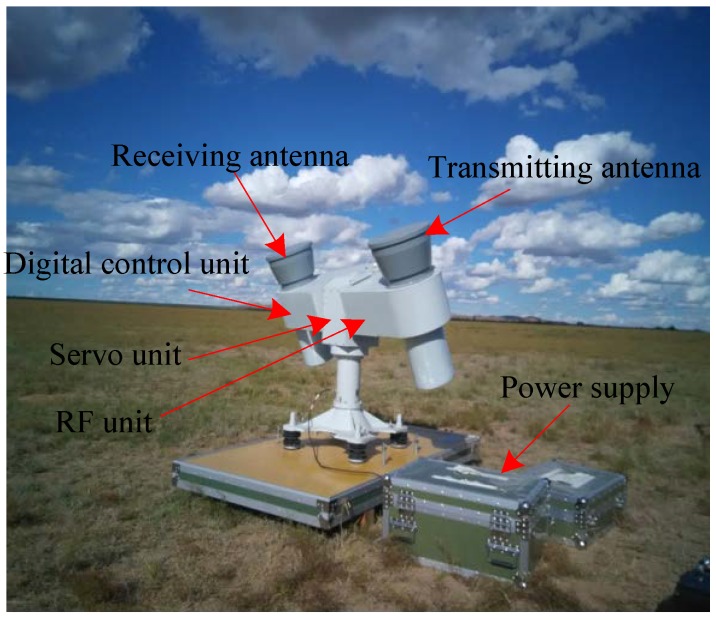
The transponder in the calibration field.

Five PARCs we developed have been used to implement the calibration campaign for the GF-3 SAR, including geometric calibration, radiometric calibration, polarimetric calibration, antenna pattern verification, the characteristic analyzing of the transmitted pulse, and so on. One of the PARCs in the calibration field is shown in Figure 2. The PARC mainly consists of five units: Antennas, servo unit, digital control unit, RF unit, and power supply. The specifications of the PARCs are listed in Table 1.

### 3.1. Antenna

**Figure 3:**
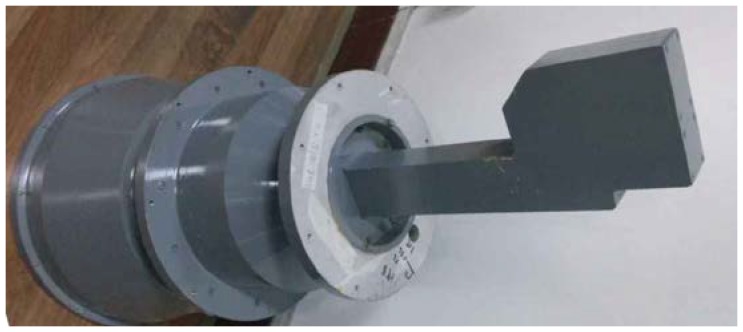
Picture of the antenna.

**Figure 4:**
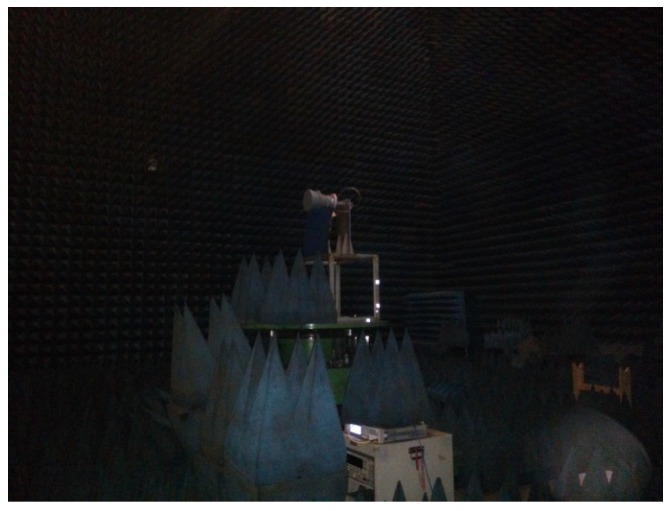
Outer view of the antenna under testing in the anechoic chamber.

**Figure 5:**
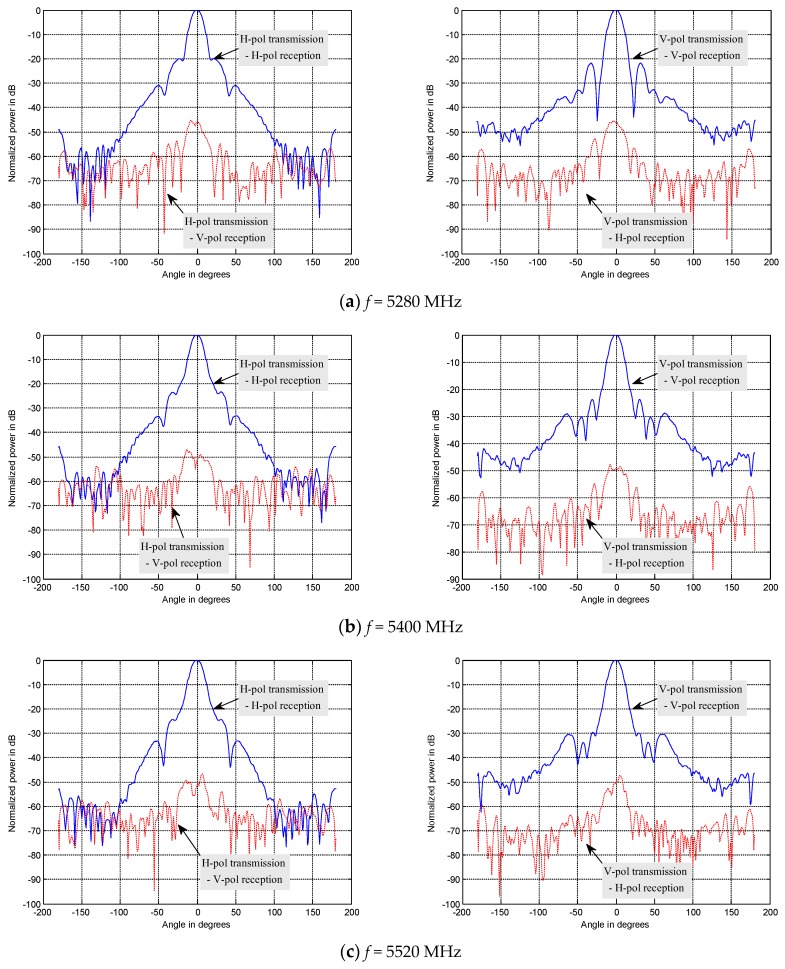
Patterns measured in the anechoic chamber (including polarization isolation).

The form and performance of the antenna decides the whole structure and performance of the PARC in some extent. We used the dual-polarimetric and high polarization isolation antenna for the first time, which simplified the structure and ensured the index of polarization isolation. The gain is about 23 dBi and the 3-dB beamwidth is about 12°, which decreases the aligning requirement between the PARC antenna and the GF-3 SAR antenna. A picture of the antenna is shown in Figure 3. The antenna can receive or transmit H and V polarization waves simultaneously, with very high polarization isolation. Figure 4 is an outer view of the antenna being tested in an anechoic chamber, and Figure 5 shows the patterns for co-polarization. We also show the cross-polarization patterns for comparison. We can see that the cross-polarization level is more than 45 dB lower than the co-polarization level in the whole frequency band of the GF-3 from 5280 MHz to 5520 MHz, which ensures the accuracy of the polarimetric calibration.

### 3.2. RF Unit

**Figure 6:**
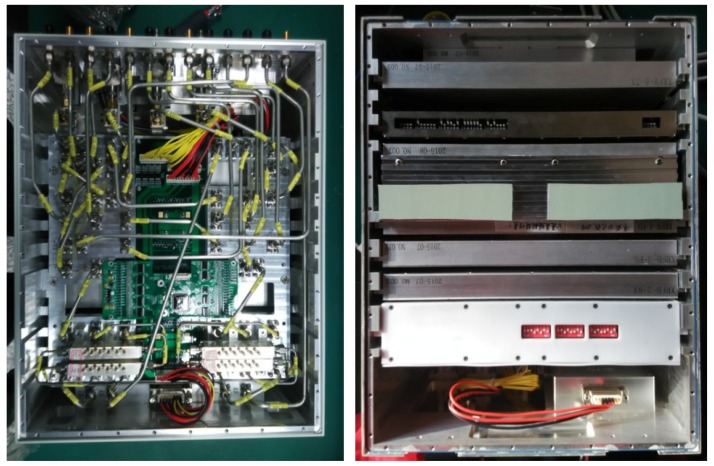
Outer view of the RF unit.

Figure 6 is an outer view of the RF unit for the PARC. It consists of the RF receiver, fiber delayer, four-pass module, gain adjustor, RF amplifier, internal calibration module, and phase shifters. The gains are about 50 dB at maximum and can be reduced by 20 dB in 5-dB steps. The center frequency is 5400 MHz, and the bandwidth is 240 MHz. Table 2 lists the characteristics of the four channels (HH/HV/VH/VV).

The chirp pulses transmitted by the GF-3 SAR travel through the atmosphere and reach the receiving antenna of the PARC. When the signal is received, on the one hand, it is recorded to analyze the characteristic of the pulse and to measure the azimuth antenna pattern, and on the other hand, it is amplified and re-transmitted to the SAR to provide a reference point. When the scattering matrix, [−1 −1; 1 1], is needed for polarimetric calibration, theoretically, both the amplitudes and phases of the HH/HV/VH/VV channels should be identical. However, although the amplitudes and phases of the four channels are adjusted to be identical in the laboratory, they maybe change when the PARC is used for an in-orbit calibration campaign due to the variation of the environment. Therefore, the special internal calibration circuit should be design to ensure the imbalance in the amplitude and phase of the HH/HV/VH/VV channels [15].

#### 3.2.1. Amplitude Calibration Circuit

**Figure 7:**
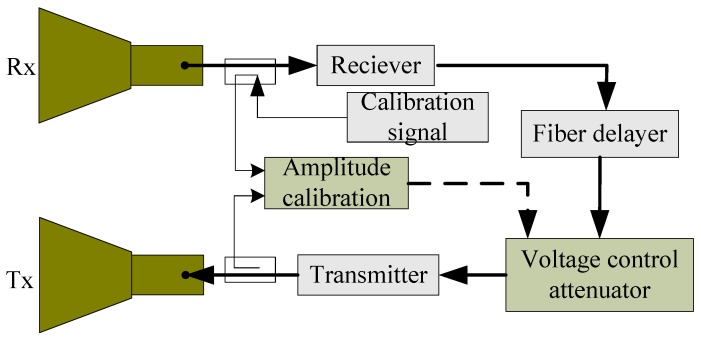
Amplitude calibration circuit scheme.

Both the amplitude stability for every channel and the imbalance in the four channels are achieved by an amplitude calibration circuit, as depicted in Figure 7. A series of calibration pulses are fed to the circuit near its input. These go around the main RF circuit, with a 2 μs or 4 μs delay, and are then detected at the amplitude calibration detector. The original pulse, without the delay, is also detected in the amplitude calibration module. The amplitudes of the delayed and direct pulses are compared, and any error nulled using the voltage control attenuator in the main RF circuit. The RF unit gain is therefore stabilized. The couplers between the amplitude calibration module, receiver/transmitter chain, and amplitude calibration module is designed in a constant-temperature box, thus, the gain stability is independent of the surroundings’ temperature. When the gains of the HH/HV/VH/VV channels are calibrated a few minutes before the GF-3 SAR passes overhead, the amplitude stability and consistency can be achieved. Through this method, the gain stability of the GF-3 PARC is ±0.1 dB and the imbalance in the amplitudes of the HH/HV/VH/VV channels is ±0.13 dB.

#### 3.2.2. Phase Calibration Circuit

**Figure 8:**
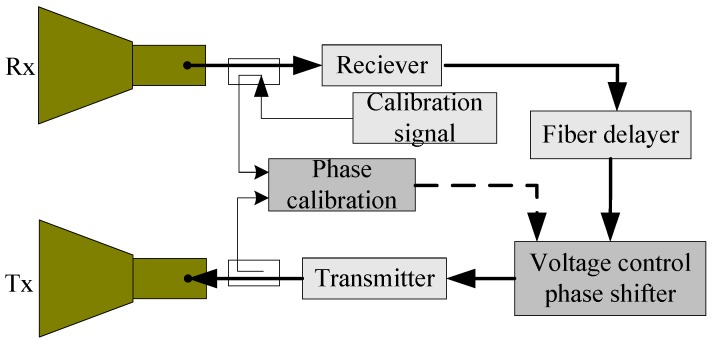
Phase calibration circuit scheme.

The GF-3 presented some new challenges to the PARC, including the phase consistency among the HH/HV/VH/VV channels, which required that the phases were almost the same and were relatively stable. We specially designed a phase calibration circuit, as illustrated in Figure 8. To allow for phase calibration, a voltage control phase shifter was included in the main RF circuit. Different from amplitude calibration, a continuous wave, in place of a pulse wave, was used as an internal calibration signal, which allowed the calibration signal travelling around the main RF circuit and the calibration signal directly going into the phase calibration module to overlap for a period. Therefore, their phases can be compared in the phase calibration circuit. By adjusting the voltage control phase shifter, the phase calibration output can be nulled, thereby the phase of the main RF circuit is the same as the phase of the reference channel. When the phases of the HH/HV/VH/VV channels are calibrated a few minutes before the GF-3 SAR passes overhead, the phase consistency can be achieved. Through this method, the phase consistency of the GF-3 PARC was up to ±2.4° with a ±1° relative phase stability.

## 4. Measurement and Experiment

### 4.1. Result of Test in Anechoic Chamber

**Figure 9:**
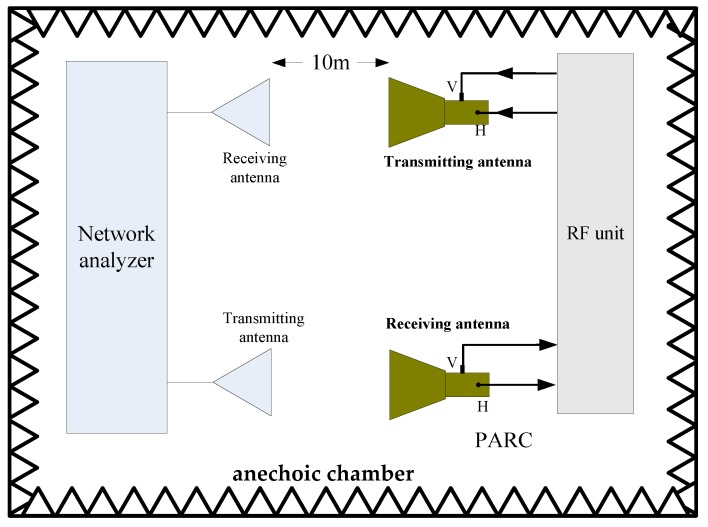
Configuration for the radar cross section (RCS) measurements.

After evaluating the antennas and RF unit, we assessed the radar cross section (RCS) characteristics of the novel PARC manufactured for the GF-3 in an aniconic chamber using two standard horn antennas and a network analyzer. Figure 9 is the configuration we used for the measurement. Two 45° rotating C-band standard gain horn antennas with a gain of 25 dB were connected to the S1 and S2 ports of the network analyzer, and we measured the S21 characteristics over the whole route, including the transmitting and receiving antennas, propagation paths, and the PARC. The transmitting and receiving polarizations of the PARC were adjusted by use of switches in the RF unit. For simplicity, we expressed the antenna polarization conditions for the PARC as a VH configuration, which is when the receiving polarization is vertical and the transmitting polarization is horizontal. The RCS of the PARC could be obtained from its *S*21 value measured as follows:

(2) $$\mathrm{PARC}\;\mathrm{RCS}=S21-10\log10(\frac{\lambda^{2}}{{\left(4\pi \right)}^{3}R^{4}})-(G_{R}-3)-(G_{T}-3)$$

where *G_R_* and *G_T_* are the gains of the receiving standard horn antenna and the transmitting standard horn antenna, respectively, and *R* is the distance. *λ* is the wavelength of the center frequency.

It can be seen for Equation (2) that when the configuration is fixed, the RCS of the PARC is only dependent on the S21 measured. The standard horn antennas were rotated by 45°, therefore, they can receive and transmit H and V polarized signals. When the antennas of the PARC and the antennas connected to the network analyzer are aligned with each other, we can control the switches in the RF unit to achieve the S21 corresponding to the HH, HV, VH, and VV channels. Table 3 gives the measurements of the RCS consistency.

From the above measurements, we proved that the present PARC has perfect imbalance with ±0.13 dB in amplitude and ±2.4° in phase, which satisfies the demands for the PARC providing the scattering matrix of [−1 −1; 1 1]. Furthermore, the undesirably polarized RCS relative to the desirably polarized RCS was less than 45 dB and the polarization purity of the present PARC is very high, which satisfies the demands for the PARC providing the scattering matrix of [0 1; 0 0] and [0 0; 1 0].

### 4.2. Result of In-Orbit Calibration

**Figure 10:**
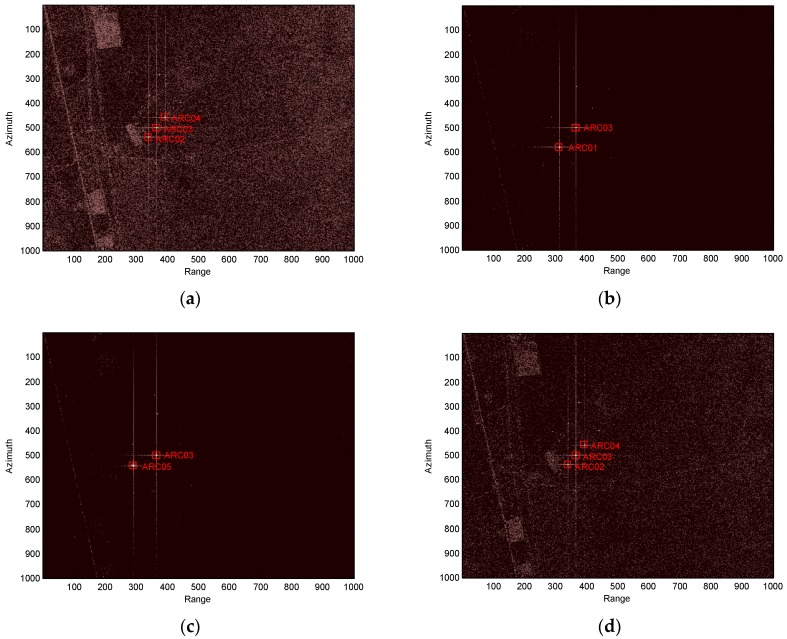
Images of the calibration field of the GF-3 for one pass. (**a**) HH image; (**b**) HV image; (**c**) VH image; (**d**) VV image.

The PARCs we developed have been utilized for the in-orbit calibration campaign of the GF-3. Next, we give the images of the PARCs for one mission. In this mission, the scattering matrices of the five PARCs are set, as shown in Table 4. Figure 10 gives the images of the calibration field obtained from the GF-3 SAR. Only ARC01 can be seen in the HV image and only ARC05 can be seen in the VH image. ARC02 and ARC04 can be seen in the HH and VV images. ARC03 can be seen in the HH, HV, VH, and VV images. This is in accordance with the set scattering matrices, as shown in Table 4.

From Figure 10, we find the amplitudes of HH, HV, VH, and VV for ARC03 are very consistent with ±0.16 dB and the polarization purity of ARC01 and ARC05 is better than 44 dB. Furthermore, the PARC images of HH, HV, VH, and HH possess good characteristic of the point target. The PARCs comfortably satisfy the GF-3 polarimetric and radiometric calibration requirements.

**Figure 11:**
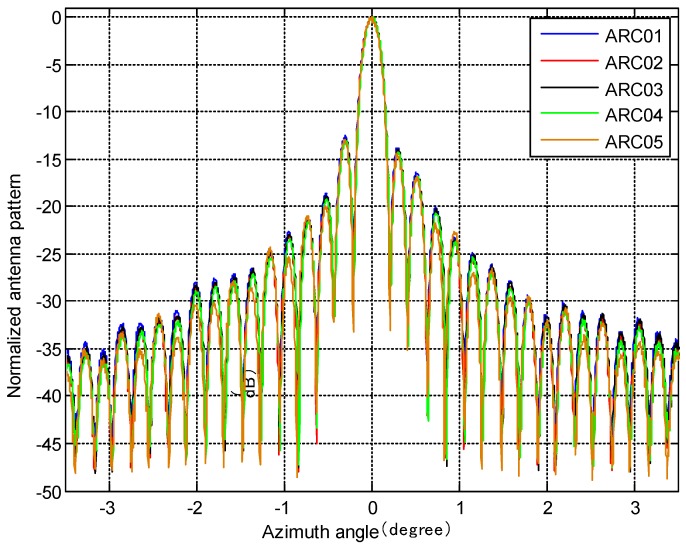
Azimuth pattern measurement for the strip-mode in quad-polarization.

Moreover, the antenna pattern in azimuth can be attained using transponder recordings [16]. The PARCs of the GF-3 SAR can detect the amplitude of the transmit pulses as a function of the time and flight movement of the GF-3 SAR. The measured azimuth antenna pattern can be compared to the pattern measured in the anechoic chamber when they are transformed to the antenna azimuth angles and corrected by the position information. For this purpose, five PARCs were deployed for one pass and each PARC was seen by the GF-3 SAR under almost the same viewing angle. The antenna azimuth pattern obtained by the PARCs is shown in Figure 11. The pattern obtained by ARC01 to ARC04 is the H-polarization antenna pattern and the one obtained by ARC05 is the V-polarization antenna pattern.

We analyzed the main lobes and peak side lobe ratio (PSLR) of the antenna pattern for every PARC. The difference within the 3-dB beam width among the patterns performed with the PARCs is less than 0.3 dB, which is due to the slight difference in the range and the slight inconsistency in the linearity for the five PARCs. The asymmetry in the azimuth pattern can be seen in Figure 11, and the first left sidelobe is about 1 dB higher than the first right sidelobe for all five patterns. It can be seen that the asymmetry really exists in the radiated azimuth antenna pattern. It also can be seen that the H-polarization azimuth antenna pattern is almost the same as the V-polarization azimuth antenna pattern.

## 5. Discussion

We developed a novel PARC used for the GF-3 that can provide many kinds of scattering matrices used to calibrate the GF-3, evaluate the image quality, and verify the calibration result. Although the novel PARC has many advantages, the system, especially the RF unit, is very complex and it is rather costly. To achieve all kinds of scattering matrices, the RF unit possess two individual RF channels to realize the four channels (HH, HV, VH, and VV), which increases the difficulty of the imbalance in the amplitude and phase, although we did realize this through the amplitude and phase internal calibration. However, the PARCs of Radarsat-2 and Sentinel-1 that use rotating antennas do not have this problem because only one RF channel is used. Of course, the rotating-antennas PARC only provides one scattering matrix for the fixed rotated antennas. Therefore, we should consider various kinds of PARC to be developed for polarimetric calibration in the future for a fully polarimetric SAR. The rotating-antennas PARC is used to provide scattering matrix of [−1 −1; 1 1] and other scattering matrices can be provided by the novel PARC presented in this paper.

In this paper, we presented the results of the PARC measured both in an anechoic chamber and in-orbit calibration. The imbalance in the amplitude and phase, and the polarization isolation were mainly discussed. We find that these indexes of in-orbit calibration are a little worse than when measured in the anechoic chamber, which is due to greater error factors and a greater abominable environment for the in-orbit calibration. Furthermore, the results of the radiometric calibration should also be discussed in a following study.

## 6. Conclusions

This paper describes the principle, design, manufacture, and, measurement results of a novel PARC used to calibrate the GF-3 SAR. Dual-polarized and high polarization isolation antennas were used in the PARC for the first time. The novel PARC we manufactured overcomes the shortcomings of the large and heavy structure of the four-antennas PARC, such as KOMPSAT-5, and the inflexible setting scattering matrix of rotated-antennas PARC, such as Radarsat-2 and Sentinel-1. The PARC was measured in the microwave anechoic chamber and took part in an in-orbit calibration. The results prove that the PARC we developed has perfect imbalance, with a better than ±0.2 dB amplitude and ±4° phase, and the polarimetric purity is better than 44 dB, which comfortably satisfies the requirements for the GF-3 polarimetric and radiometric calibration.

## Figures and Tables

**Table 1:** Specifications of the polarimetric active radar calibrator (PARC).

Parameter	Value
Frequency	C-band
Radar cross section (RCS)	40~60 dBsm
RCS step	5 dB
Polarization Isolation	More than 40 dB
RCS stability	0.2 dB
Antenna pointing precision	Better than 0.2°
Amplitude consistency among HH/HV/VH/VV channels	0.4 dB (peak to peak)
Phase consistency among HH/HV/VH/VV channels	8° (peak to peak)

**Table 2:** Characteristics of the four channels at 5400 MHz. PDBC: Phase difference between the four channels (based on the HH channel).

	HH Channel	HV Channel	VH Channel	VV Channel
Gain	50.1 dB	50.02 dB	49.92 dB	50.18 dB
PDBC	0 degree	−4.8 degree	−3.8 degree	−4.7 degree

**Table 3:** RCS consistency measurement of PARC.

Configuration	S21/dB	S21/degree	RCS/dBsm
HH	5.92	84.3	60.00
HV	5.84	79.5	59.92
VH	5.74	80.5	59.82
VV	6.00	79.6	60.08

**Table 4:** Scattering matrix setup.

PARC	ARC01	ARC02	ARC03	ARC04	ARC05
Scattering Matrix	$\left[\begin{array}{ll} 0 & 1\\ 0 & 0 \end{array}\right]$	$\left[\begin{array}{ll} 1 & 0\\ 0 & 1 \end{array}\right]$	$\left[\begin{array}{rr} -1 & -1\\ 1 & 1 \end{array}\right]$	$\left[\begin{array}{ll} 1 & 0\\ 0 & 1 \end{array}\right]$	$\left[\begin{array}{ll} 0 & 0\\ 1 & 0 \end{array}\right]$
